# Effect of phosphate group on remineralization of early enamel caries regulated by amelogenin peptide

**DOI:** 10.1371/journal.pone.0303147

**Published:** 2024-05-21

**Authors:** Hualei Zhao, Qun Zhang, Jinpu Chu

**Affiliations:** 1 The First Affiliated Hospital of Zhengzhou University, Zhengzhou, China; 2 Zhengzhou Stomatological Hospital, Zhengzhou, China; International Medical University, MALAYSIA

## Abstract

**Objective:**

To show the effect of the phosphate group on the remineralization process of early enamel caries mediated by amelogenin peptide.

**Methods:**

Freshly extracted, completed, and crack-free bovine teeth were used to create artificial early enamel caries, which were randomly divided into four groups: Group A: fluorination remineralized solution treatment group; Group B: pure remineralized solution treatment group. Group C: 100 g/ml recombinant Amelogenin peptide remineralized solution treatment group (with single phosphate group on N-terminus); Group D: 100 g/ml non-phosphorylated recombinant Amelogenin peptide remineralized solution treatment group (without single phosphate group on N-terminus). For 12 days, fresh remineralized solutions were replaced daily. Transverse microradiography (TMR) was used after remineralization to determine mineral loss and demineralization depth before and after each sample’s remineralization. Each sample’s depth of remineralization and mineral acquisition were then determined.

**Results:**

The recombinant amelogenin peptide group significantly outperformed the non-phosphorylated amelogenin peptide group in terms of mineral acquisition and mineralization depth (P<0.05).

**Conclusions:**

The recombinant Amelogenin’s solitary phosphate group at the N-terminus helps recombinant Amelogenin to encourage the remineralization process of early enamel caries.

## 1. Background

Dental caries is a chronic progressive disease caused by alternating demineralization and remineralization accumulations [1]. An essential natural restorative process, known as enamel remineralization, involves the repair of partially dissolved enamel crystals by the deposition of calcium and phosphorus ions in saliva [2]. But still, the pure deposition crystal does not possess the same higher-order structure as natural enamel.

Using scanning electron microscopy, Kwak et al. demonstrated that amelogenin has the ability to promote the growth of enamel crystals with higher-order structure similar to sound enamel [3]. From the perspective of enamel crystal formation, Amelogenin’s functional fragments are the basis of biomimetic remineralization materials for enamel, they share the following characteristics: 1) stabilize the calcium and phosphate ions of solution, and inhibit spontaneous precipitation; 2) bind to amorphous calcium phosphate (ACP) to form a nanocomplex with high affinity for the enamel crystal surface; and 3) provide a nucleation site or mineralization template for the orderly formation of crystals [4–8]. We obtained recombinant amelogenin peptide in earlier studies and demonstrated its capacity to remineralize enamel, particularly in the subsurface lesion body of early enamel caries [9]. We also discovered that: 1).Tested by transmission electron microscopy and selected area electron diffraction, this peptide could interact with calcium ions in the solution and stabilize ACP; 2).The optimal concentration of amelogenin peptide for remineralization is 100 g/ml [9]. So We think this recombinant amelogenin will be able to bind with calcium ions to stabilize ACP and enter the lesion body of early enamel caries through enamel pores [9]. The recombinant amelogenin peptide functions as a "ion carrier" in the remineralization of early enamel caries.

According to research [10], the solitary phosphate group is present at the Ser-16 location at the highly conserved N-terminal of amelogenin. This single phosphate group has the potential to bind to ACP and impact protein-mineral interactions [11–14]. Wiedemann-Bidlack et al. also discovered that the solitary phosphate group in amelogenin plays a key role in the process of ACP stabilization [15–17]. Moreover, mineral deposition and the quality of ACP are related during the remineralization process.

The single phosphate group exists at the N-terminal of recombinant amelogenin. In order to explore the influences of single phosphate group on amelogenin-mineral interaction and remineralization effect, we used phosphorylated amelogenin and non-phosphorylated amelogenin to carry out the remineralization experiments of artificial early enamel caries in vitro. In contrast to our prior study, the main research object of this experiment is the single phosphate group on the N-terminus of recombinant amelogenin, whereas the previous research object is recombinant amelogenin [9]. By examining the mineral deposition in the lesion body, the impact of a single phosphate group on the remineralization controlled by amelogenin was identified. If our hypothesis is right, the non-phosphorylated amelogenin group will be unable to bind with ACP and so will be unable to transport ACP to the lesion location to complete the remineralization process. It suggests that the remineralization impact of non-phosphorylated amelogenin is less than that of phosphorylated amelogenin. We established a positive control group and a negative control group to observe the remineralization impact of non-phosphorylated amelogenin.

## 2.Methods

### 2.1. Enamel specimen preparation and peptide synthesis

Extracted bovine teeth were collected according to guidelines approved by the Ethics Committee of the first affiliated Hospital of Zhengzhou University. Enamel discs (3 mm × 3 mm × 2 mm)were obtained from the cleaned buccal surfaces of bovine incisors. Under stereoscopic microscope, the specimens which have observable cracks, white spot lesions, or enamel malformation were removed. The superficial enamel surfaces of the discs were then serially polished with water-cooled silicon-carbide discs (320, 600, 800, 1200, 1500, 2000, and 2500 grade of Al2O3 paper; Buehler Ltd.),removed the outermost enamel~150μm, deionized water rinsing in an ultrasonic device for 10 minutes, we finally got enamel specimens with uncontaminated enamel surfaces. For each specimen, we divided 3 mm × 4 mm buccal window into two windows of 3 mm × 2 mm (*a* and *b*). All surfaces of each specimen were coated with acid-resistant nail varnish, except for buccal windows *a* and *b*. Window *a* served as control site, while window *b* served as the experiment site [18].

The phosphorylated peptide, comprising the first 45 amino acid residues of the N-terminus (which contains a phosphate group on serine 16) and the last 11 residues of the C-terminus of porcine amelogenin, was synthesized by Syn-peptide Co., Ltd., (Shanghai, China) using standard solid-phase peptide synthesis, purified and identified using HPLC and ESI-MS, which up to standard. The non-phosphorylated peptide was prepared from phosphorylated peptide by incubation with alkaline phosphatase. Lyophilized peptide was dissolved in Millipore-purified water at 200μg/mL and stored at 4°C. Peptide stock solutions were centrifuged (10,900 × g, 4°C,20 min) just before use.

### 2.2. Caries lesion formation

At a constant temperature of 37°C, 40 enamel specimens were exposed to demineralization solution [2.2 mM Ca(NO_3_)_2_, 2.2 mM KH_2_PO_4_,50 mM CH_3_COOH, 1 mM NaN_3_, 0.5 mM NaF. 3ml per specimen]for 3 days with magnetic stirring at 100 RPM [19]. The pH was adjusted to 4.5 with KOH solution(Sigma, St.Louis, MO, United States). Each enamel specimen was rinsed carefully with sufficiently distilled deionized water and air-dried after the demineralization. Window *a* was coated with acid-resistant nail varnish as a baseline control.

### 2.3. Remineralization testing

The enamel specimens were randomly divided into 4 groups(10 specimen/group) with the following treatment: Group A: Fluorinated remineralized solution; Group B: pure remineralized solution; Group C: 100μg/ml phosphorylated peptide remineralized solution; Group D: 100 μg/ml non-phosphorylated peptide remineralized solution. The fluorinated remineralized solution for Group A, was prepared by adding fluoride to the pure remineralized solution, with a fluoride concentration of 2ppm. The pure remineralized solution for Group B was prepared as previously reported[1.5 mM CaCl_2_, 0.9 mM KH_2_PO_4_, 130 mM KCL, 1 mM NaN_3_, 20 mM HEPES] [9]. The remineralized solution for Group C and Group D were prepared by adding 100μg/ml phosphorylated peptide and 100μg/ml non-phosphorylated peptide to the pure remineralized solution respectively. The pH was adjusted to 7.0 with KOH for all groups. All treatments were performed at 37°C.All reagents were purchased from Sigma-Aldrich (Sigma, St.Louis, MO, United States).

Specimens were put into corresponding remineralized solutions (1.5 ml per specimen) [9] at 37°C for 12 days under continuous magnetic stirring(100RPM). Remineralized solutions were replaced every day. After remineralization, the enamel blocks were cleaned with sufficient deionized water and air-dried.

### 2.4. TMR test

The specimens of Group A-D were embedded in epoxy resin(ZM-AB-2,China).With buccal windows facing upwards and sliced vertically, several initial specimen-slices were obtained from each specimen. By polishing with hand-polish machine and silicone-carbide paper under water-cooling, we ended up with specimen-slices of each group as thin as 120μm. All specimen-slices contained both *a* and *b* sections. The TMR images of specimen-slices were taken with an aluminum step wedge, using the Softex X-ray system(Softex, Tokyo, Japan) to measure the progression of enamel demineralization. Exposure setting was 30min at 20kv, 20mA. The TMR images were digitized using a digital camera(Canon, Japan) connected to an Optical microscope(ZEISS, Germany). The demineralization area (section *a*)and remineralization area(section *b*) of each specimen-slice were selected for analysis. Mineral loss (kg/m^2^) and lesion depth (μm) were calculated using the measured mineral content (vol%) of the specimen-slices [20], considering the maximum mineral content of the sound (non-treated) enamel to be 87vol% and the mineral density to be 3156 kg/m^3^. Mineral acquirement (kg/m^2^) was also calculated as the mineral loss in section *a* minus the mineral loss in section *b*. Similarly, the depth change value was also calculated as the lesion depth in section *a* minus the lesion depth in section *b* [18].The remineralization capacity of each group was evaluated based on the mineral acquirement and the depth change value.

### 2.5. Data analysis

Remineralization data was analyzed with SPSS 21.0 (IBM, Chicago, IL, United States) using α = 0.05. The mineral acquirement and depth change value among groups were analyzed by One-way ANOVA and LSD-t test for multiple comparisons.

## 3. Results

**Fig 1:**
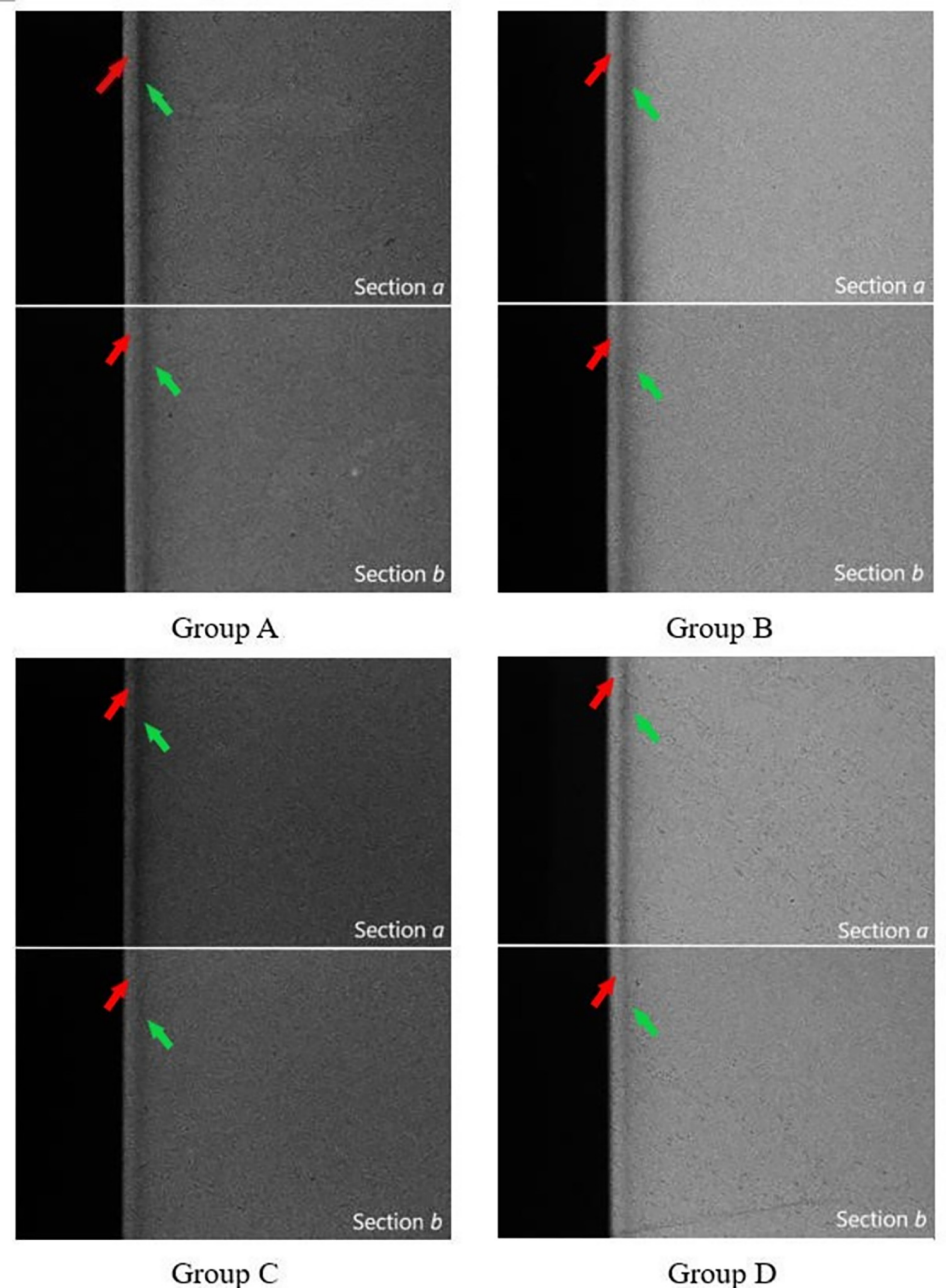
TMR images. Section a: demineralization area, the control site. Section b: remineralization area, the test site. The red arrow points to the relatively complete enamel surface, the green arrow points to the subsurface demineralized area. the lesion body density in each group generally increased, indicating that both groups exhibited the remineralization effect.

A total of 40 specimens were prepared and analyzed, with 10 specimens for each remineralized solution treatment group. Fig 1 shows an example of enamel X-ray projection images for each group. The light surface bands on each image, indicated by the red arrow, correspond to the relatively complete enamel surface of early enamel caries. The subsurface area marked with the green arrow is less dense and corresponds to the lesion body of early enamel caries. After remineralization treatment, lesion body density generally increased in each group(section b, the experimental site), indicating that both groups exhibited the remineralization effect.

**Fig 2:**
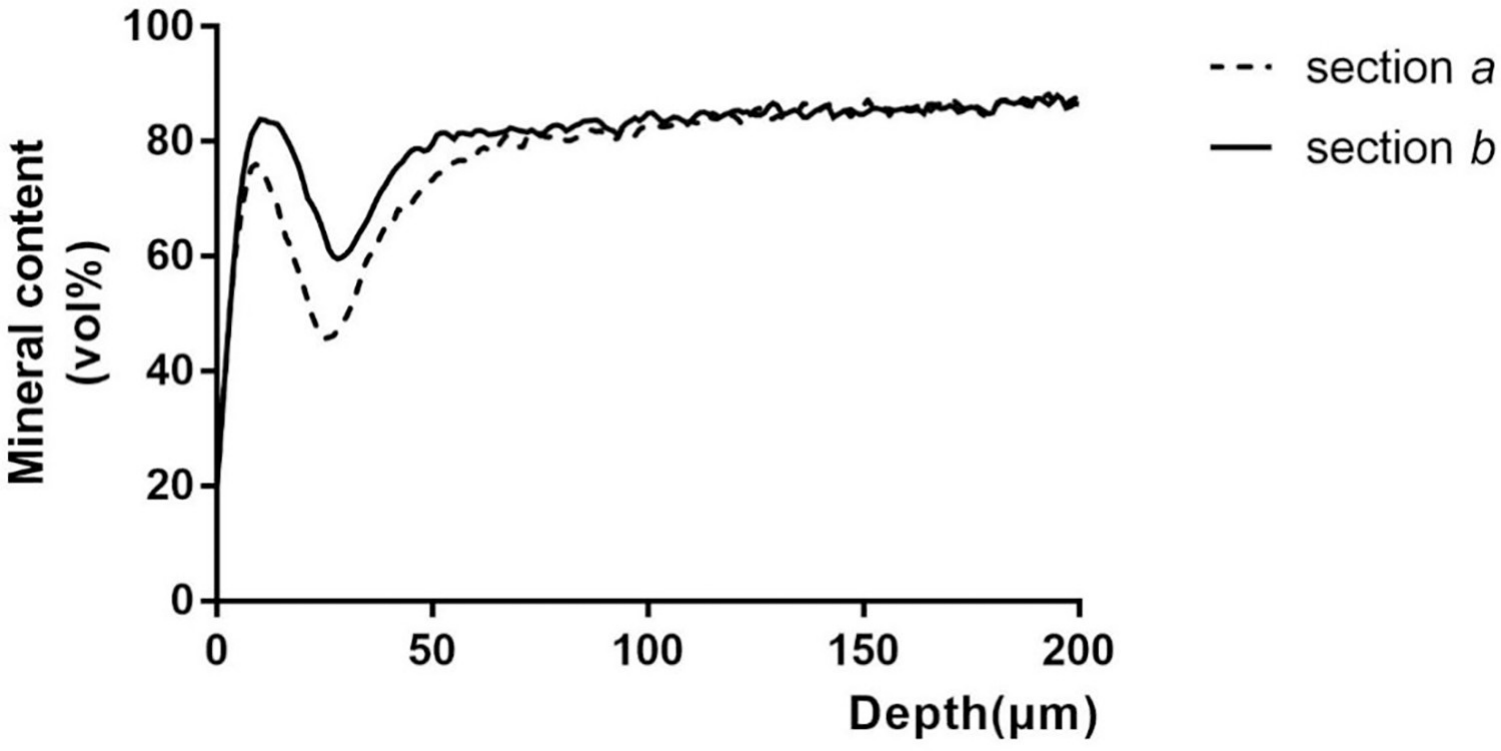
Mineral content (vol%)—depth (m). Section a: demineralization area, the control site. Section b: remineralization area, the experimental site. The first upward curve represents the mineral content of the sample enamel surface. Subsequent downward and upward curves indicate that the degree of demineralization gradually increases in the area of the subsurface lesion, after reaching the lowest point, the mineral content gradually returns to normal. The non-coincident portion of two curves showing the remineralization effect of the experimental solution.

**Fig 3:**
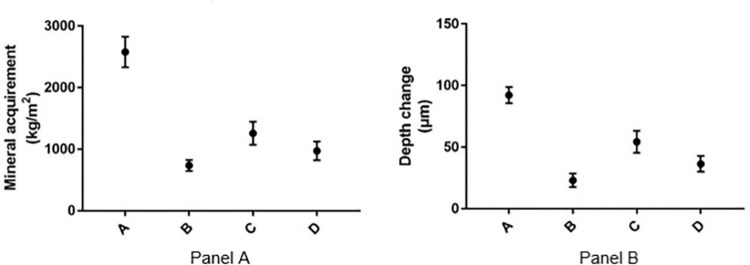
Mineral acquirement(kg/m2) and depth change(μm).(Mean±SD). A: Fluorinated remineralized solution; B: pure remineralized solution; C: 100μg/ml phosphorylated peptide remineralized solution; D: 100 μg/ml non-phosphorylated peptide remineralized solution. P<0.05.

Fig 2 shows the correlation between mineral content and depth in early enamel caries in a specimen. The mineral content curve can be divided into three areas: surface, lesion body and sound enamel. In the surface area, the mineral content gradually increases to its peak, which represents the mineralized surface of early enamel caries after demineralizing and remineralizing cycles. In the lesion body, the mineral content decreases from the highest point to the lowest point and then slowly increases showing the change in mineral content with depth of the lesion. Eventually, the curve tends to be stable as the mineral content of the lesion body gradually increases close to sound enamel. In Fig 2, the area between the demineralization curve and the remineralization curve shows mineral acquirement. Mineral acquirement and depth change were calculated for each sample and the results for each group are presented in Fig 3. Panel A shows the mean and standard deviation of mineral acquirement for four different groups and Panel B shows depth change values. Group D’s mineral acquisition and depth change were significantly lower than Group A(p<0.05), significantly higher than Group B(p<0.05),and significantly lower than Group C (p<0.05).

## 4. Discussion

In vivo, the initial enamel crystals form from the assembly and fusion of ACP particles. The different facets of nano hydroxyapatite crystallites could be connected by ACP so that the assemblies could become more flexible. And the assemblies could also maintain a better chemical continuity of the formed mineral as the ACP could transform into hydroxyapatite [21, 22]. The single phosphate group present at the N-terminus of amelogenin could bind to ACP and affect protein-mineral interactions during enamel development in vivo [11–14]. In previous studies, we verified the remineralization ability of recombinant amelogenin. In order to investigate the effect of each phosphate group in the remineralization process and to provide a theoretical basis for the biomimetic remineralization of tooth enamel, we developed this experiment.

This study demonstrates that the single phosphate group peptide can promote remineralization process in early enamel caries. Four remineralization treatments were applied to four groups of specimens and the measured mineral acquirement and depth change indicated that specimens treated with phosphorylated peptide showed a stronger remineralization effect compared to specimens treated with non-phosphorylated peptide. without the single phosphate group, recombinant amelogenin’s ability to promote mineral deposition in caries area was decreased. This study shows that the single phosphate group peptide can promote the remineralization process in early enamel caries. Four groups of samples underwent four remineralization treatments, measured mineral acquirement and depth change showed that samples treated with phosphorylated peptide exhibited a greater remineralization effect than samples treated with non-phosphorylated peptide. Without the single phosphate group, the ability of the recombinant amelogenins to promote mineral deposition in the carious area was reduced.

In a previous study we obtained the recombinant amelogenin peptides and verified their ability to mineralize, including binding to calcium ions, thereby stabilizing ACP and inhibiting the premature formation of calcium phosphate crystals in solution, and now we found that this ability is related to the single phosphate group at the N-terminus of this recombinant peptide amelogenin [9]. This is a finding consistent with the research by Kwak et al. Furthermore, we found that different concentrations of phosphorylated amelogen peptide proteins showed different abilities to stabilize ACP.

The phosphorylation state of amelogenin have a direct impact on amelogenin-mineral interactions and the regulation of mineralization processes [9]. Combined with previous studies,the ability of phosphorylated amelogenin to stabilize ACP is positively correlated with the mineralization effect. The initial enamel crystals form from the assembly and fusion of ACP particles [21].The different facets of nano hydroxyapatite crystallites could be connected by ACP hence the assemblies could be more flexible. Since the ACP could transform into hydroxyapatite, the assemblies could also maintain better chemical continuity of the formed mineral than the mesocrystals [22].That is, as a precursor to mineralization, the amount of ACP entering the lesion area is critical to the remineralization effect.

The phosphorylated amelogenin could not only adhere to the enamel surface, but also penetrate the interior of early enamel caries through demineralized enamel pores. In this experiment, we investigate the effect of phosphate group on early enamel caries remineralization regulated by amelogenin peptide in vitro and showed the positive influence of phosphate group on mineral deposition in the lesion body of early enamel caries. TMR was used to determine the mineral loss and lesion depth of each specimen before and after remineralization, and then we calculated the mineral acquirement and depth change. The remineralization effect of four groups was compared by analyzing the mineral acquirement and depth(lesion depth) change of each group. TMR data from four groups showed that the remineralization effect of recombinant amelogenin was significant than that of pure remineralized solution but still weaker than that of fluorinated remineralized solution(p<0.05). The remineralization effect of recombinant amelogenin was better than non-phosphorylated amelogenin, suggesting that the phosphate group could help recombinant amelogenin promote remineralization of early enamel caries. The phosphate group act as an”ACP delivery system” and promote mineral deposition in the lesion area of early enamel caries. As we all know, mineral deposition is the first step of enamel crystal formation. This suggests that we may be able to enhance the remineralization effect by using phosphate groups to promote mineral deposition. However, the remineralization effect of non-phosphorylated amelogenin was still more significant than that of pure remineralized solution(p<0.05). This indicates that the single phosphate group is not the only factor promoting remineralization of early enamel caries regulated by recombinant amelogenin. We must say, as in vitro experiment, we still have limitations. Regarding time pressure and uncertainty about the expected results, only the intuitive remineralization effect was observed, lack molecular-level interaction research.

In a word, the phosphorylated amelogenin is likely propelled by the concentration gradient, diffusing through the porous structure of demineralized enamel and eventually reaching the subsurface lesion area.After this synthetic peptide transported ACP to the subsurface lesion area of early enamel caries, ACP was converted to hydroxyapatite crystals. In the remineralization process, the single phosphate group at the N-terminus of recombinant amelogenin is essential for mineral deposition.

## 5. Conclusion

The phosphate group at the N-terminus of recombinant amelogenin may promote mineral deposition in the subsurface lesions of early enamel caries. In combination with our previous studies, we believe that the recombinant amelogenin binds with calcium ions and stabilizes ACP through the phosphate group. And then the recombinant amelogenin-ACP complex enters the lesion body of early enamel caries through enamel pores. After firmly bonding with hydroxyapatite (HA), the ACP continues the crystallization process, turning into hydroxyapatite crystals and completing the remineralization process on the surface of HA in the lesion area. These results remind us that we may be able to enhance amelogenin’s remineralizing action through the phosphate group.

## Supporting information

S1 File (PDF)

S1 Data (XLSX)
