# Supplementary material for: Effect of phosphate group on remineralization of early enamel caries regulated by amelogenin peptide
Source: PLoS One. 2024 May 21;19(5):e0303147. doi: 10.1371/journal.pone.0303147 (PMC11108222; doi:10.1371/journal.pone.0303147)
Supplement: S1 File — (PDF) [file pone.0303147.s001.pdf]

```
ONEWAY 矿物获得量 深度变化 BY 组别
/STATISTICS DESCRIPTIVES HOMOGENEITY
/MISSING ANALYSIS
/POSTHOC=LSD ALPHA(0.05).
```

单向

[数据集1] D:\工作\文章\plos one\数据\数据处理2019\完整.sav

描述

		N	均值	标准差	标准误	均值的 95% 置信区间		极小值
						下限	上限	
矿物获得量	1	7	2578.57	246.538	93.183	2350.56	2806.58	2220
	2	7	735.71	88.291	33.371	654.06	817.37	630
	3	7	1258.57	186.764	70.590	1085.84	1431.30	1000
	4	7	972.86	150.523	56.892	833.65	1112.07	780
	总数	28	1386.43	744.880	140.769	1097.59	1675.26	630
深度变化	1	7	92.343	6.5800	2.4870	86.257	98.428	85.7
	2	7	23.086	5.5001	2.0789	17.999	28.172	17.1
	3	7	54.400	9.0170	3.4081	46.061	62.739	42.5
	4	7	36.471	6.4619	2.4424	30.495	42.448	27.7
	总数	28	51.575	27.3170	5.1624	40.983	62.167	17.1

描述

		极大值
矿物获得量	1	2890
	2	850
	3	1590
	4	1180
	总数	2890
深度变化	1	101.6
	2	32.8
	3	70.6
	4	45.5
	总数	101.6

方差齐性检验

	Levene 统计量	df1	df2	显著性
矿物获得量	1.421	3	24	.261
深度变化	.401	3	24	.753

单因素方差分析

		平方和	df	均方	F	显著性
矿物获得量	组间	14224157.14	3	4741385.714	150.384	.000
	组内	756685.714	24	31528.571		
	总数	14980842.86	27			
深度变化	组间	18968.293	3	6322.764	128.636	.000
	组内	1179.660	24	49.153		
	总数	20147.953	27			

在此之后检验

多重比较

LSD

因变量	(I) 组别	(J) 组别	均值差 (I-J)	标准误	显著性	95% 置信区间 下限	95% 置信区间 上限
矿物获得量	1	2	1842.857*	94.911	.000	1646.97	2038.74
		3	1320.000*	94.911	.000	1124.11	1515.89
		4	1605.714*	94.911	.000	1409.83	1801.60
	2	1	-1842.857*	94.911	.000	-2038.74	-1646.97
		3	-522.857*	94.911	.000	-718.74	-326.97
		4	-237.143*	94.911	.020	-433.03	-41.26
	3	1	-1320.000*	94.911	.000	-1515.89	-1124.11
		2	522.857*	94.911	.000	326.97	718.74
		4	285.714*	94.911	.006	89.83	481.60
	4	1	-1605.714*	94.911	.000	-1801.60	-1409.83
		2	237.143*	94.911	.020	41.26	433.03
		3	-285.714*	94.911	.006	-481.60	-89.83
深度变化	1	2	69.2571*	3.7475	.000	61.523	76.992
		3	37.9429*	3.7475	.000	30.208	45.677
		4	55.8714*	3.7475	.000	48.137	63.606
	2	1	-69.2571*	3.7475	.000	-76.992	-61.523
		3	-31.3143*	3.7475	.000	-39.049	-23.580
		4	-13.3857*	3.7475	.002	-21.120	-5.651
	3	1	-37.9429*	3.7475	.000	-45.677	-30.208
		2	31.3143*	3.7475	.000	23.580	39.049
		4	17.9286*	3.7475	.000	10.194	25.663
	4	1	-55.8714*	3.7475	.000	-63.606	-48.137
		2	13.3857*	3.7475	.002	5.651	21.120
		3	-17.9286*	3.7475	.000	-25.663	-10.194

*. 均值差的显著性水平为 0.05。
